# Supplementary material for: Polyketide synthase gene clusters in the ericoid mycorrhizal fungus Oidiodendron maius: genome-wide prediction, expression profiling, and functional characterization of knockout mutants
Source: Front Plant Sci. 2026 Sep 16;17:1924430. doi: 10.3389/fpls.2026.1924430 (PMC13623903; doi:10.3389/fpls.2026.1924430)
Supplement: Supplementary file 1 [file SupplementaryFile1.zip › Supplementary Materials/Supplementary Folder 1.zip!/Oidma1_AssemblyScaffolds_Repeatmasked/knownclusterblast/cluster54/jgi.p_Oidma1_94684_mibig_hits.html]

| MIBiG Protein | Description | MIBiG Cluster | MiBiG Product | % ID | % Coverage | BLAST Score | E-value |
| --- | --- | --- | --- | --- | --- | --- | --- |
| AIA58901.1 | putative\_WD-40\_protein | BGC0001141 | Brefeldin\_biosynthetic\_gene\_cluster | 53 | 98.8372093023 | 93 | 1.4e-19 |
| AMH40424.1 | WD40\_repeat-containing\_protein | BGC0001350 | Phormidolide\_biosynthetic\_gene\_cluster | 46 | 70.9302325581 | 66 | 1.4e-11 |
| CAF34034.1 | putative\_WD-repeat-containing\_protein | BGC0000689 | 2-deoxystreptamine\_biosynthetic\_gene\_cluster | 53 | 67.4418604651 | 54 | 5.6e-08 |
| CAF31443.1 | putative\_gentamicin\_production\_protein | BGC0000696 | Gentamicin\_biosynthetic\_gene\_cluster | 53 | 67.4418604651 | 54 | 5.6e-08 |
| AAR98560.1 | GntN | BGC0000697 | Gentamicin\_biosynthetic\_gene\_cluster | 53 | 67.4418604651 | 54 | 5.6e-08 |
| ACN38354.1 | putative\_WD-repeat-containing\_protein | BGC0000714 | Sisomicin\_biosynthetic\_gene\_cluster | 52 | 67.4418604651 | 52 | 3.6e-07 |
